# Complete or partial loss of the Y chromosome in an unselected cohort of 865 non-vasectomized, azoospermic men

**DOI:** 10.1186/s12610-023-00212-z

**Published:** 2023-12-14

**Authors:** J Fedder, C Fagerberg, MW Jørgensen, CH Gravholt, A Berglund, UB Knudsen, A Skakkebæk

**Affiliations:** 1 Centre of Andrology & Fertility Clinic, Odense University Hospital, Kløvervænget 23, DK-5000 Odense, Denmark; 2 Department of Clinical Medicine, University of Southern Denmark, Odense, Denmark; 3 Fertility Clinic, Horsens Hospital, Horsens, Denmark; 4 Department of Clinical Genetics, Odense University Hospital, Odense, Denmark; 5 Department of Clinical Genetics, Lillebaelt Hospital, Vejle, Denmark; 6 Department of Molecular Medicine, Aarhus University Hospital, Aarhus, Denmark; 7 Department of Endocrinology, Aarhus University Hospital, Aarhus, Denmark; 8 Department of Clinical Medicine, Aarhus University, Aarhus, Denmark; 9 Department of Clinical Genetics, Aarhus University Hospital, Aarhus, Denmark

**Keywords:** Azoospermia, Y chromosome loss, 45,X/46,XY mosaicism, Y microdeletion, Y chromosome, Azoospermie, Perte du Chromosome Y, Mosaïque 45,X/46,XY, Microdélétions Y, Chromosome Y

## Abstract

**Background:**

Structural abnormalities as well as minor variations of the Y chromosome may cause disorders of sex differentiation or, more frequently, azoospermia. This study aimed to determine the prevalence of loss of Y chromosome material within the spectrum ranging from small microdeletions in the azoospermia factor region (AZF) to complete loss of the Y chromosome in azoospermic men.

**Results:**

Eleven of 865 azoospermic men (1.3%) collected from 1997 to 2022 were found to have a karyotype including a 45,X cell line. Two had a pure 45,X karyotype and nine had a 45,X/46,XY mosaic karyotype. The AZF region, or part of it, was deleted in eight of the nine men with a structural abnormal Y-chromosome. Seven men had a karyotype with a structural abnormal Y chromosome in a non-mosaic form. In addition, Y chromosome microdeletions were found in 34 men with a structural normal Y chromosome. No congenital malformations were detected by echocardiography and ultrasonography of the kidneys of the 11 men with a 45,X mosaic or non-mosaic cell line.

**Conclusions:**

In men with azoospermia, Y chromosome loss ranging from small microdeletions to complete loss of the Y chromosome was found in 6.1% (53/865). Partial AZFb microdeletions may give a milder testicular phenotype compared to complete AZFb microdeletions.

## Résumé

**Contexte:**

Des anomalies structurelles ainsi que des variations mineures du chromosome Y peuvent provoquer des troubles de la différenciation sexuelle ou, plus fréquemment, une azoospermie. Cette étude visait à déterminer la prévalence de la perte de matériel chromosomique Y dans le spectre allant de petites microdélétions dans la région du facteur d’azoospermie (AZF) à la perte complète du chromosome Y chez les hommes azoospermiques.

**Résultats:**

Onze des 865 hommes azoospermiques (1,3 %), collectés entre 1997 et 2022, présentaient un caryotype comprenant une lignée cellulaire 45,X. Deux avaient un caryotype pur 45,X et neuf avaient un caryotype mosaïque 45,X/46,XY. La région AZF, ou une partie de celle-ci, était absente chez huit des neuf hommes présentant un chromosome Y anormal sur le plan structurel. Sept hommes présentaient un caryotype avec un chromosome Y structurellement anormal sous une forme non mosaïque. De plus, des microdélétions du chromosome Y ont été trouvées chez 34 hommes présentant un chromosome Y de structure normale. Aucune malformation congénitale n’a été détectée par échocardiographie et échographie des reins des 11 hommes porteurs d’une lignée cellulaire 45,X mosaïque ou non mosaïque.

**Conclusions:**

Chez les hommes qui ont une azoospermie, une perte du chromosome Y, allant de petites microdélétions à une perte complète du chromosome Y, a été observée chez 6,1 % (53/865). Les microdélétions partielles de la région AZFb peuvent donner un phénotype testiculaire plus doux que les microdélétions complètes de l’AZFb.

## Introduction

The male has a heterogametic sex in majority of mammals, including humans, and has a monofactorial sex-determining mechanism based on an XX/XY sex chromosome system [1]. Due to reduced recombination the Y chromosome has lost 97% of genes over time and as a consequence decreased in size [2, 3]. Sequencing the human Y chromosome has been difficult due to a high number of rearrangements, inversions, duplications and deletions [4]. Thus, the Y chromosome shows extensive complexity and variation between men [5].

The Y chromosome contains the sex-determining region Y (SRY) gene on the short arm (p arm) [6] and the azoospermia factor (AZF) region with spermatogenesis-related genes on the long arm (q arm) [7, 8]. Structural abnormalities as well as minor variations of the Y chromosome may cause disorders of sex differentiation or, more frequently, azoospermia. Structural abnormalities of the Y chromosome are relatively frequent and include partial or complete deletions of the long or short arm, Y isochromosomes, isodicentric Y chromosomes, and ring Y chromosomes [9].

**Fig. 1:**
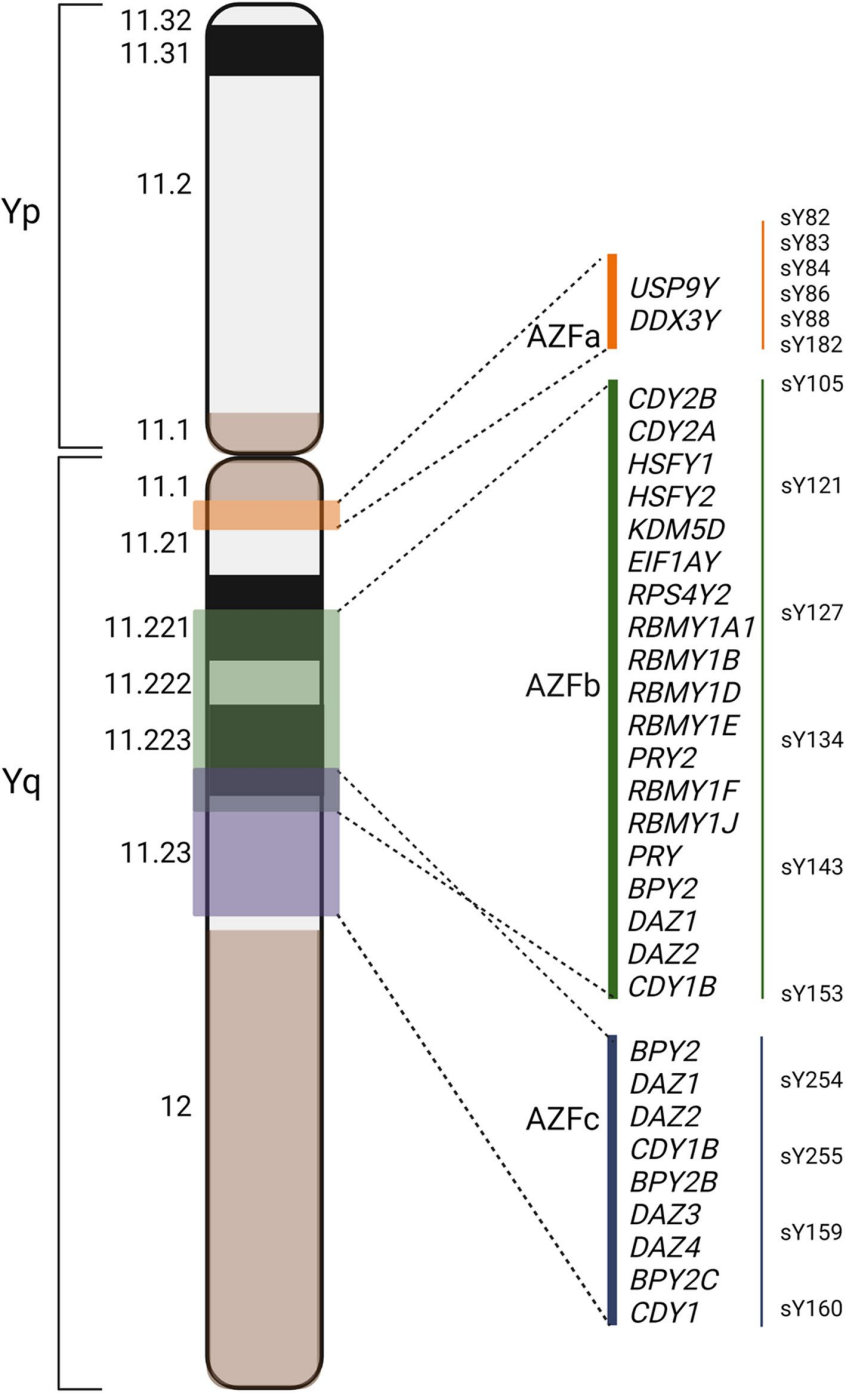
Schematic illustration of the human Y chromosome. The azoospermia factor (AZF) regions on the long arm of the Y chromosome: AZFa, AZFb and AZFc contain genes important for spermatogenesis

The AZF region are important for spermatogenesis, and deletions including this region results in azoospermia or oligozoospermia. Based on their breakpoints AZF deletions are classified into: AZFa (the smallest microdeletion located closest to the centromere), AZFb and AZFbc (the larger ones), and AZFc (including the most distal Yq region close to the heterochromatin region) [10] (Fig.1). The region earlier termed “Deleted-in-AZoospermia” (DAZ) is located in the AZFc region [11].

A genetic abnormality can be found in at least 1/3 of men with azoospermia [12, 13]. Among chromosomal aberrations, particularly Klinefelter syndrome have a high prevalence [14, 15]; nonetheless, a wide spectrum of other, more rare, chromosomal aberrations are also found. These include 45,X/46,XY mosaicism and variants hereof, or more rarely, azoospermic men with a pure 45,X karyotype in the blood. Patients with a pure 45,X karyotype are usually phenotypic females [16], unless the SRY gene has been translocated to another chromosome.

In patients with mosaicism for a 45,X cell line and a cell line including Y chromosome, a particularly high prevalence of structural variants of the Y chromosome is found, likely because an abnormal Y chromosome is unstable and thus has risk of being lost during cell division [9].

In this study, we present data from an unselected cohort of 865 azoospermic men who went through the same examination program, which allows evaluation of the prevalence of men with complete or partial Y chromosome loss among men with azoospermia.

## Patients and methods

### Patients

Since 1997, we have consecutively included non-vasectomized, azoospermic men referred to our andrology center and fertility clinics into the present cohort. From 1997 to April 2011 the patients were examined at Fertility Clinic, Braedstrup (Horsens) Hospital, and from May 2011 at Centre of Andrology and Fertility Clinic, Odense University Hospital. At least two ejaculates without sperm in raw semen as well as in the pellet obtained after centrifugation were required for a diagnosis of azoospermia. The patients make up the majority of azoospermic men referred for fertility treatment in Western Denmark for about 25 years [12].

### Clinical examination, including ultrasonography of the scrotum

All men underwent our routine examination programme, which included a detailed medical history interview and physical examination as previously described [12]. Scrotal ultrasonography was performed according to Fedder [17]. Since we do not have a real control group, testicular volumes of patients with Y loss were compared with testicular volumes of azoospermic men with *CFTR* variants and congenital bilateral absence of vas deferens (CBAVD) shown to have intact spermatogenesis [12]. Blood samples were obtained for analysis of hormone levels and genetic status, including analyses for *CFTR* variants. Hormones analysed included follicular stimulating hormone (FSH) and luteinizing hormone (LH), testosterone, prolactine (reference interval: (73-411) mIU/L), and thyroid stimulating hormone (TSH; reference interval: (0.30-4.0) mIU/L)). In addition, inhibin-B, anti-müllerian hormone (AMH; reference interval: (5.5-103) pmol/L)), and estradiol (reference interval: (0.04-0.16) nmol/L) have been measured since 2014 [12]. Further reference intervals are given in Table 2.

The testosterone analysis was based on protein precipitation with deuterium marked components, and testosterone concentration calculated from the proportion of deuterium marked molecules compared to non-marked molecules. Inhibin B was analysed by a specific two-sided enzyme immunometric assay (Beckman Coulter Ltd.). Further hormones were determined using electrochemiluminescense immunoassays.

### Karyotyping

**Fig. 2:**
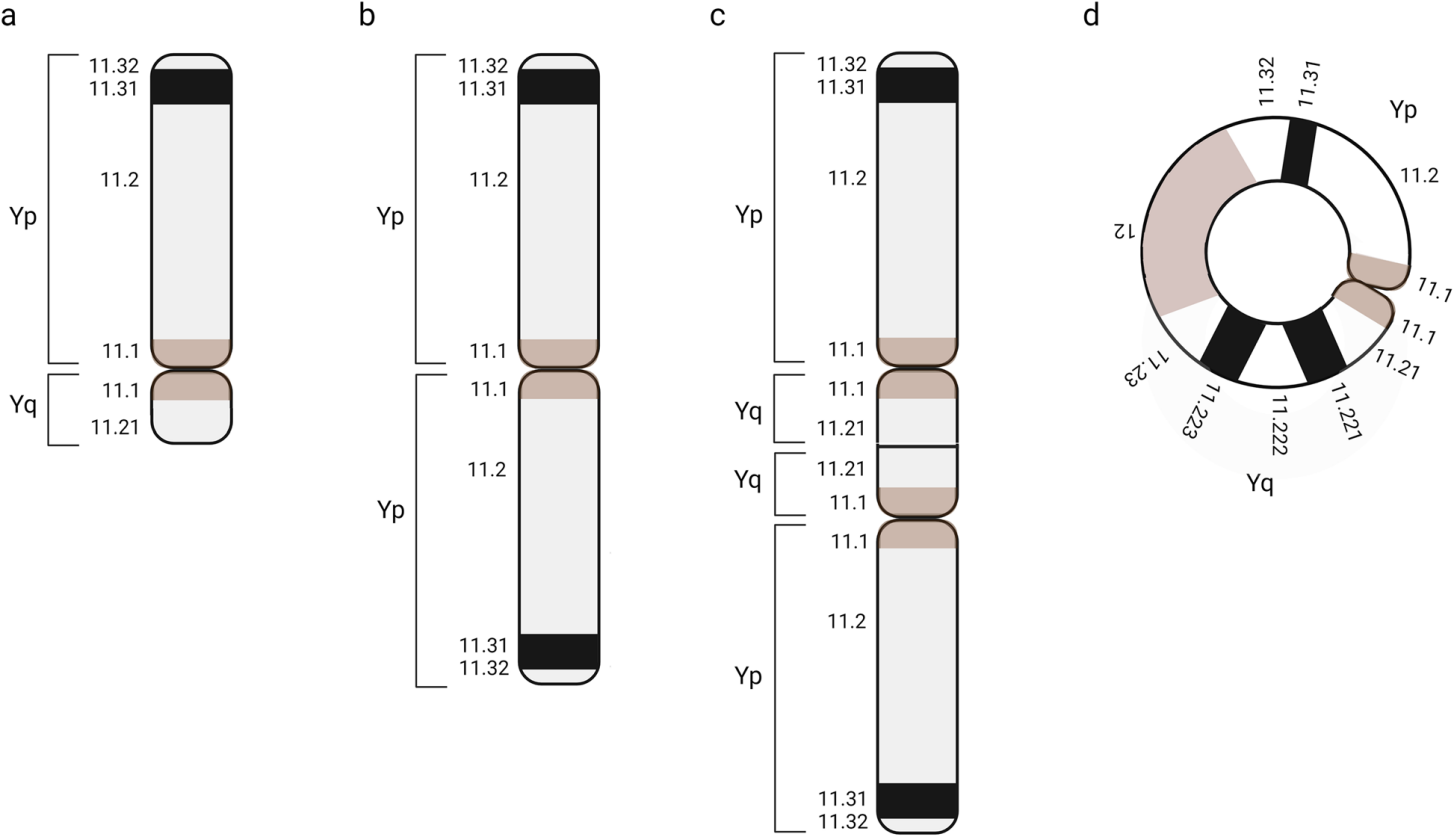
The most common structural Y chromosome abnormalities are: **a** deletions of the long arm of the Y, **b** Y isochromosomes, **c** isodicentric Y chromosomes, and **d** Y ring chromosomes

Chromosome analysis was performed on cultured peripheral blood lymphocytes using standard methods. At least 10 metaphases were examined for each sample as a standard. When mosaicism was suggested, a larger number of cells were counted. In addition fluorescence in situ hybridization (FISH) screening with relevant probes (i.e. SRY targeted probes) was done when relevant. Abnormal Y chromosomes were classified as illustrated in Fig. 2.

### Analysis of AZF microdeletions

DNA was extracted from peripheral blood samples using standard methods, and analysed for Y microdeletions encompassing specific AZF-regions using recommended polymorphic markers and a multiplex PCR technique [6]. According to recommendations from the European Academy of Andrology (EAA) and European Molecular Genetics Quality Network (EMQN), two markers were routinely used for each AZF region: AZFa (SY84, SY86), AZFb (SY127, SY134) and AZFc (SY254, SY255). In selected cases, the analysis was extended with analysis for more markers: AZFa (SY82, SY83, SY88, SY1182), AZFb (SY105, SY121, SY143, SY153) and the heterochromatic region just distal to AZFc (SY160 and sometimes SY159).

Until 2014 the analysis was supplemented with the polymorphic markers SY152 (proximal in the AZFc region) and SY157 (distal in the AZFc region), when the polymorphic markers SY254 and SY255 were absent. However, because sY152 is also mapping to other genomic regions [8], it has been excluded from the analysis since 2014.

### Transthoracic echocardiography (TTE) and Renal Ultrasonography (RUS)

Men with a 45,X cell line were referred for TTE and RUS at local hospitals due to an increased prevalence of congenital heart and kidney malformations in female 45,X patients [18]. The examinations were performed according to local guidelines.

### Testicular biopsy

Testicular biopsy was considered in all cases, but men with Klinefelter syndrome were recommended microdissection-TESE (m-TESE) or, in a period, alternatively unilateral testicular orchiectomy [19] without testicular biopsy in advance. Testicular biopsy was performed as described previously, usually using a TruCut needle [12, 20]. Histological patterns were categorised into normal spermatogenesis, maturation arrest, hypospermatogenesis, and Sertoli-cell-only syndrome, as described in detail by Fedder et al*.* [12].

### Statistical analysis

Comparison of prevalence’s of abnormal Y chromosomes in men with and without 45,X cell lines was performed using Fisher’s exact test. A two-tailed t-test was used for comparison of testicular volumes and FSH values in men with 45,X, 45,X/46,XY and variants hereof and/or AZF microdeletions compared with men with congenital bilateral absence of vas deferens (CBAVD) combined with pathogenic *CFTR* variants.

## Results

The mean age of the cohort was 32.4 years of age (18y-58y), and 85% (735/865) were of ethnically Danish origin.

**Fig. 3:**
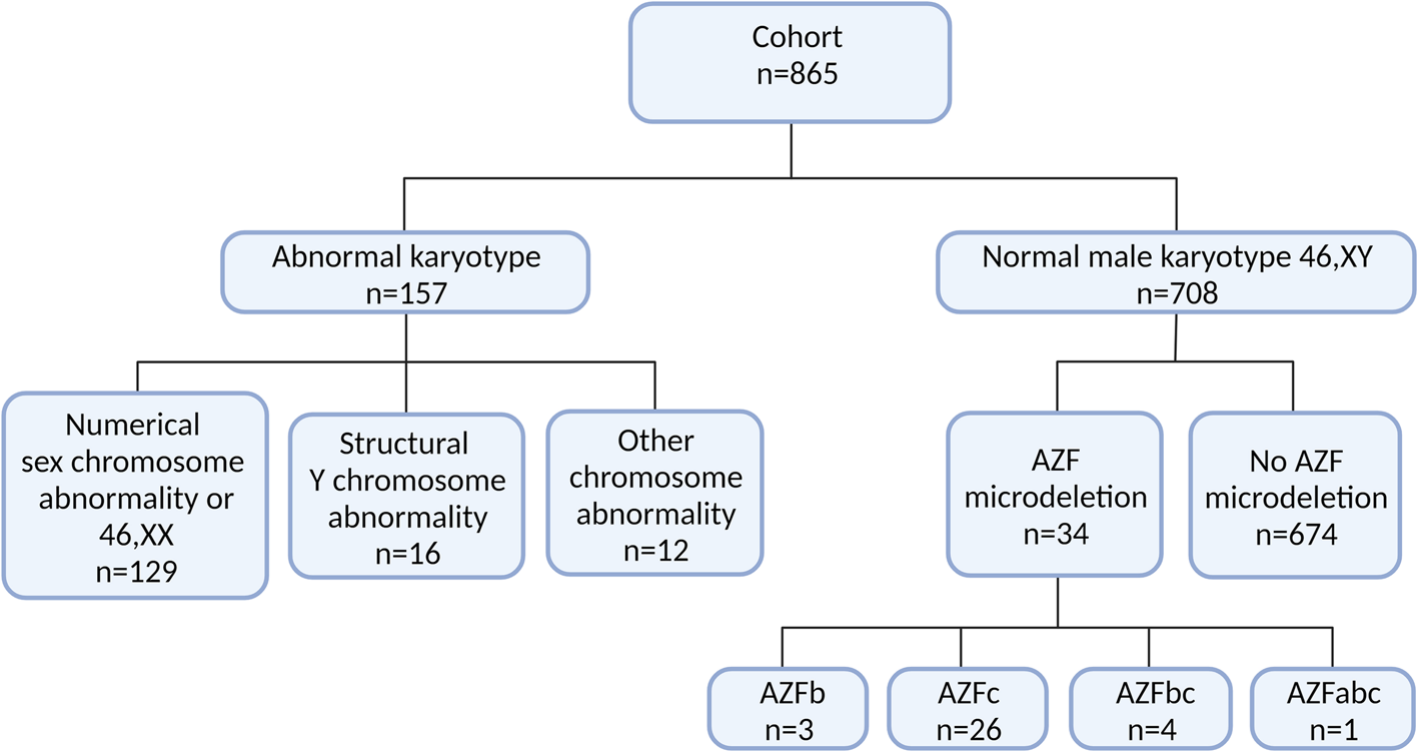
Illustration of karyotype abnormalities and Y microdeletions detected in the cohort of 865 consecutive, azoospermic men

Among the 865 azoospermic men, 157 (18.2%) were found to have a numerical or structural chromosome abnormality (Fig. 3). Numerical sex chromosome abnormalities were seen in 129 men (14.9%) with Klinefelter syndrome or mosaics hereof being the most frequent karyotype seen (120 men (13.9%), with 112 men (12.9%) having a non-mosaic 47,XXY karyotype). Other chromosomal abnormalities such as 46,XX, 47,XYY and autosomal translocations, or translocations involving the X chromosome each made up less than 1% (Table 1).

**Table 1:** Association between chromosome abnormalities and Y microdeletions in a cohort of 865 azoospermic men

**Karyotypes of patients**	**Number of patients**	**Missing AZF regions**			
		**AZFabc**	**AZFbc**	**AZFb**	**AZFc**
**Numerical sex chromosome abnormalities including 46,XX**					
45,X	2	2	-	-	-
46,XX	3	3	-	-	-
47,XXY	112	-	-	-	-
47,XXY mosaicism	8	-	-	-	-
47,XYY	3	-	-	-	-
48,XXYY	1	-	-	-	-
**Chromosome abnormalities with Y chromosome deletions**					
45,X/46,X,del(Y)(q11.1)	1	1	-	-	-
45,X/46,X,del(Y)(q11.21)	1	-	1	-	-
45,X/46,X,del(Y)(?)	1	-	1	-	-
46,X,del(Y)(q11.2)	1	1	-	-	-
46,X,del(Y)(q11.22)	1	-	1	-	-
46,X,del(Y)(q11.23)	2	1	1	-	-
46,X,del(Y)(q?)	1	-	1	-	-
**Chromosome abnormalities with an isochromosome Y**					
45,X/46,X,i(Y)(q11)	2	-	2	-	-
45,X/46,X,del(Y)/46,X,i(Y)	1	-	(1)	-	-
45,X/46,X,idic(Y)(q11)	1	-	1	-	-
45,X/46,X,idic(Y)(q11.222)	1	-	1	-	-
46,X,idic(Y)(q11.2)	1	-	1	-	-
**Other Y chromosome abnormalities**					
45,X/46,X,r(Y)	1	-	-	-	-
46,X,der(Y),dup(Y),del(Y)	1	-	-	-	-
**X:autosomal translocation**	2				
**Y:autosomal translocation**	1				
**Autosomal translocation**	5				
**Autosomal inversion**	3	-	-	-	-
**Small supernumerary chromosome**	1				
**46,XY**	708	1	4	3	26

Eleven men (1.3%) were found to have a karyotype including a 45,X cell line. Two had a pure 45,X karyotype with SRY translocated to chromosome 14 and chromosome 21, respectively, and nine had a mosaic karyotype with a cell line with 45,X and a 46,XY cell line with a structural abnormal Y chromosome (Table 1). Among the nine men with mosaic karyotype and a structural abnormal Y chromosome the following structural abnormalities of the Y chromosome were detected: deletion of majority of the long arm, Y isochromosome, isodicentric Y chromosome, and ring Y chromosome. One man had three cell lines, with two of them presenting a structural abnormal Y chromosome (45,X/46,X,del(Y)/46,X,idic(Y) (Table 1).

Among men with a structural abnormal Y chromosome without a 45,X cell line (*n*=7), six had a partial Y chromosome deletion (in one case combined with a partial Y chromosome duplication), and one had an isodicentric Y chromosome. Three men had a 46,XX karyotype (Table 1). In two of these 46,XX males, the SRY gene was translocated to one of the X chromosomes, while SRY could not be detected in the third 46,XX male.

In the present cohort of 865 azoospermic men, deletions of the majority of the long arm of the Y chromosome, Y isochromosomes, isodicentric Y chromosomes with duplication of the short arm, and Y ring chromosomes were represented in all nine men with 45,X/46,XY mosaicism but only in seven of the 854 cases without a 45,X cell line (*p*<0.00001; Fisher’s exact test), showing that the frequency of Y chromosome loss is higher in men with Y chromosome abnormalities.

Except for two men with a ring Y chromosome and a combination of Y duplication and Y deletion, and two men with a Yq deletion, the AZFb and AZFc regions were not detected in any of the men with a structural abnormal or missing Y chromosome. Furthermore, the AZFa region was missing in both 45,X men, the three with 46,XX karyotype, three with Yq-deletion, and in one man with 46,XY karyotype (Table 1).

Among the 843 azoospermic men with one or more karyotypically normal Y chromosome 4.0% (*n*=34) had an AZF microdeletion. AZFc microdeletion were detected in 26 men (3.1%), whereas AZFb, AZFbc and AZFabc were seen in 3, 4 and 1 patient, respectively (Table 1). In the total cohort, parts of the AZF region was missing in 53 (6.1%), including five men without a Y chromosome (Table 1).

Men with a karyotype including a 45,X cell line (pure 45,X karyotype or 45,X/46,XY mosaicism) had a mean height of 174.1 cm and a mean weight of 77.9 kg, and reduced testicular volumes (mean volume, right: 5.1 ml; left: 5.1 ml) (Table 2). Men with a pure 45,X karyotype or a karyotype with a 45,X mosaicism or AZF microdeletion usually presented with reduced testicular volumes and elevated FSH levels compared to azoospermic men with pathogenic *CFTR* variants and CBAVD (Table 3). None of the men with Y chromosome loss, including AZF microdeletions, had pathologically high numbers of small, testicular hyperechogenic foci (former termed testicular microlithiasis) [17].

**Table 2:** Height, weight, testis volumes, and FSH, LH and testosterone serum levels for the 11 patients with a 45,X cell line. The AZFb+c regions were deleted in all cases except for the man with a Y ring. Reference values for the hormones given in brackets, and hormone levels written in bold are outside the reference interval. “-” represents the absence of a value

**Karyotype**	**n**	**Height (cm)**	**Weight (kg)**	**Testis dxt. mL)**	**Testis sin (mL)**	**FSH (1.1-7.9) IU/L**	**LH (1.5-11) IU/L**	**Testosterone (8.4-30) nmol/L**
45,X	2	173 / 169	66 / 75	5.6 / 6.2	5.9 / 4.9	*18* / *11*	5.0 / 4.5	12.8 / *6.3*
45,X/46,X,del(Y)	3	- / 184 / 180	- / 80 / 72	3.6 / 4.0 / 4.2	2.2 / 5.0 / 5.4	*10.8* / *13* /*26*	7.8 / 5.2 / 8.3	11.3 / 10.4 / 13.2
45,X/46,X,i(Y)	2	182 / 188	98 / 116	2.3 / 6.5	2.7 / 5.5	*8.5* / *21*	6.6 / *17*	10.7 / 12.3
45,X/46,X,idic(Y)	2	- / 168	- / 68	- / 4.4	- / 4.4	*30* / *11*	*12* / 3.5	11.6 / 12.3
45,X/46,X,r(Y)	1	161	67	6.9	9.1	3.1	2.5	*6.9*
45,X/46,X,del(Y)/46,X,i(Y)	1	162	59	6.8	5.4	3.8	4.0	8.8

**Table 3:** Testicular volumes (both testicles) and FSH values for azoospermic men with complete Y chromosome loss or minor Y microdeletions compared with men with pathogenic CFTR mutations, CBAVD and normal spermatogenesis. The differences calculated using two-tailed t-test

**Genetic diagnosis**	**N**	**Testicular volume (mL)**		**FSH (IE/L)**	
		**Mean ± SD**	***p*-value**	**Mean ±SD**	***p*-value**
46,XX	3	4.7 ± 3.1 (*n*=3)	*p*<0.01	25.7 ± 15.5 (n=3)	*p*<0.0001
45,X	2	11.3 ± 0.3 (*n*=2)	NS	14.5 ± 4.4 (*n*=2)	*p*<0.0001
45,X/46,XY mosaicism	9	9.8 ± 3.6 (*n*=8)	*p*<0.001	14.1 ± 9.5 (*n*=9)	*P*<0.001
46,XY; AZFc missing (-sperm in testis)	17	14.6 ± 8.2 (*n*=17)	*P*<0.001	12.7 ± 6.6 (*n*=16)	*P*<0.00001
46,XY; AZFc missing (total)	26	14.5 ± 7.6 (*n*=25)	*P*<0.0001	12.1 ± 5.8 (*n*=24)	*P*<0.00001
46,XY; AZFc missing (+sperm in testis)	7	14.6 ± 6.9 (*n*=7)	*P*<0.01	9.7 ± 3.6 (*n*=6)	*P*<0.001
46,XY; AZFb missing	3	18.2 ± 4.1 (*n*=3)	NS	5.9 ± 1.2 (*n*=3)	NS
^a^CFTR mut. + CBAVD (control)	21	33.3 ± 17.0 (*n*=21)	-	4.4 ± 2.3 (*n*=21)	-

^a^Published in Fedder et al., 2021 [12]

Thirty men with loss of Y chromosome material, including 20 men with an AZFc microdeletion, had a histological evaluation based on a testicular needle biopsy (Table 4). Sertoli-cell-only syndrome (SCOS) was found in three cases with an AZFabc microdeletion, while hypospermatogenesis was found in three of six men with an AZFbc deletion, and the other three had SCOS (Table 4). In the three men with SCOS the AZFbc microdeletion was complete, while a partial microdeletion of the AZFb region was found in one man with 45,X/46,XY mosaicism, AZFbc microdeletion, and a histological pattern showing presence of spermatogonia and spermatocytes. In the man with a partial deletion of the AZFb region, sY105 was detected while sY127, sY134, sY121 and sY143 were absent suggesting that the proximal region of AZFb was present. Furthermore, in one man with an isolated AZFb microdeletion and hypospermatogenesis with presence of spermatogonia, primary and secondary spermatocytes and a few spermatids (but no spermatozoa) the sY105 and sY153 were present, while sY127, sY134, sY121 and sY143 were absent, suggesting that also this man might have a partial AZFb microdeletion.

**Table 4:** Histological pattens in 30 azoospermic men with AZF microdeletions from the cohort undergoing testicular needle biopsy

**AZF deletion type**	**Normal spermatogenesis**	**Hypospermatogenesis**	**Maturation arrest**	**Sertoli-cell-only syndrome**
**AZFabc**	-	-	-	3
**AZFbc**	-	3	-	3
**AZFb**	-	1	-	-
**AZFc**	-	^a^15	1 (late)	4

^a^Five men showed SCOS in >95% of the testicular tissue evaluated

In another AZFbc microdeleted man with hypospermatogenesis and the presence of spermatogonia and spermatocytes the AZFbc markers: sY105, sY121, sY127, sY134, sY143, and sY153 were all absent. Unfortunately, the third AZFbc microdeleted man with hypospermatogenesis, who was included early in the programme, was only analysed for the two basis markers in each region, and both AZFb markers s127 and sY134 were absent.

Among the 20 histologically evaluated men with only AZFc microdeletions, 15 presented with hypospermatogenesis, although SCOS was found in >95% of the testicular tissue samples evaluated in five cases (Table 4).

All 11 men with a pure 45,X karyotype or mosaicism for 45,X underwent TTE and RUS. These examinations were in all cases normal, except for one man (45,X/46,XY,del(Y)(q11.21)) in whom a 2.7 cm cyst was detected at the lower pole of the right kidney. Supplementary CT scan revealed that it was a simple cyst not requiring treatment. All presented normal TSH and prolactin values.

## Discussion

The present study on consecutively referred azoospermic men reflects the great variety of causes that underlie azoospermia. It highlights the importance of a standardized approach to the patient and that karyotyping still plays a central role in making the right diagnosis with additional help from molecular genetics.

Given that approximately 1% of the adult male population has azoospermia [21], a frequency of men with karyotypically abnormal or missing Y chromosomes may be 1:4000 (22/865) and a 45,X cell line may be present in 1:8000 (11/865) with approximately 1.3% of azoospermic men carrying a 45,X cell line, as found in this study. A Danish study has suggested a frequency of 45,X/46,XY mosaicism and variants hereof of 1:15.000 [22]. However, these suggestions may be somewhat imprecise being based on only 865 azoospermic men and only 34.000 newborn children, respectively. Also Patsalis et al. [23] discovered a high prevalence of Y chromosome deletions in men with sex chromosome mosaicism.

A frequency of azoospermic men with a 45,X cell line of 1.3% is much higher than a frequency of Y chromosome loss of 0.05% in blood cells in boys less than 15 years of age, but similar to a frequency of 1.34% found in 76-80 years old men from the background population [24]. However, the situation in the present study is quite different, since the older men with 45,X/46,XY mosaicism have a Y chromosome without a structural abnormality, while the present Y chromosome is usually abnormal in the younger azoospermic men. This abnormal Y chromosome forms the background for development of 45,X/46,XY mosaicism and at the same time explains why the men usually do not produce sperm.

While men with 45,X/46,XY mosaicism are not extremely rare, only three men with a pure 45,X karyotype, including the two men in this study, are registered in the Danish Cytogenetic Central Register (personal communication), which includes all karyotypes analysed in Denmark since 1967 [25].

### Mosaicism

Monocentric isochromosomes where the two arms are mirror images of each other, may be due to abnormal division of the centromere or translocation of homologous chromosomes [26]. Isodicentric chromosomes may be formed early in embryogenesis through nondisjunction of deleted Y chromosomes [27] or crossing over between palindromes, or recombination between Yq palindromes on sister chromatids [26]. In isodicentric chromosomes usually only one centromere is active, particularly if the intercentromeric distance is long [26]. Ring Y chromosomes are formed by fusion of deleted Y chromosome ends or fusion of telomeres or subtelomeric regions without chromosomal loss [28].

The finding that a mosaic karyotype including a 45,X cell line is frequently seen in combination with a structural abnormal Y chromosome is expected, as structural abnormal chromosomes are unstable [9]. Furthermore, sex chromosomes may be lost from blood cell lines with increasing age [29–31]*.* It has even been suggested that all women with Turner syndrome may possess some degree of mosaicism since it has been found that ~99% of embryos or fetuses with pure 45,X karyotypes do not survive until birth [16, 31].

Several studies have described cases with more than one cell line with an abnormal Y chromosome in addition to a 45,X cell line [27, 32] as found in one case in the present study. Thus, simultaneous occurrence of more than one cell line with an abnormal Y chromosome seems higher than expected by chance. It has been proposed that nondisjunction of Y chromosomes with deletions may result in cell lines with isodicentric Y chromosomes as well as 45,X cell lines [27].

Y chromosome loss may be accelerated by smoking [33] and has been found to correlate with the risk of cancer [34] and Alzheimer’s disease [35]. It can be difficult to know whether Y chromosome loss is the cause or the consequence of cancer development. However, genes on the Y chromosome has been found to have a tumor suppressor role [36]. Since the Y chromosome may be easier to live without, Y chromosome loss are more frequent than loss of other chromosomes [37]. The mechanisms how Y chromosomes are lost are not fully understood. However, centromeres may be defective so chromosome duplicates are not correctly segregated, and furthermore chromosomes located away from the other chromosomes of the cell may be isolated in micronuclei. And it has been shown that non-disjunctioned sister Y chromosomes more often than expected by chance are incorporated into micronuclei – and therefore lost in future cell generations. Furthermore, Y chromosomes seem to have a faster telomere shortening than other chromosomes, and chromosomes with short telomeres more probably are incorporated into micronuclei [36].

### 45,X and 46,XX males

The *SRY* gene is the master sex determining factor in most mammals [3]. *SRY* is suggested to have evolved from a *SOX3*-similar gene on ancestral sex chromosomes [3]. The Y chromosome usually shrinks during evolution due to lack of recombination [38], and in mammal species such as mole vole and spiny rat, an X0 sex chromosome constitution has been found [39, 40], and SRY has not been detected in these species [38]. One possible explanation for this apparently lack in *SRY* may be due to lack in access due to the vast variation in *SRY* between species [3]. If *SRY* is absent in these few mammal species, another master sex determining factor may be present [41, 42].

In the two patients with 45,X karyotype, the *SRY* gene was translocated to chromosome 21 and chromosome 14, respectively. This is parallel to a sex-reversed mouse model, where the SRY is translocated to an autosome as a transgene. With this mouse model it has been possible to generate mice with a gonadal sex independent of the sex chromosome constitution. This has given researchers the possibility to study the influence of sex chromosomes on for example autoimmune diseases [43] and adiposity [44]. However, since major differences in gene expression, X-Y crossing-over, and X chromosome inactivation exist in humans and mice [45], studies in 45,X men with the *SRY* translocated to an autosome may provide a foundation for learning more about the role of sex chromosomes in disease pathogenesis in humans.

Translocations of Y chromosome material to autosomes such as chromosome 14 [46] and chromosome 21 [47] are very rarely described. In the case described by Petit et al. [46], a small piece of the long arm of chromosome Y was translocated to chromosome 14. The study was conducted before the detection of *SRY* and the *AZF* region, but the *SRY* gene was most likely intact in this man, who had azoospermia. In the case described by Hillman et al*.* [47], the short arm, the centromere, and the proximal part of the long arm of the Y chromosome were completely missing, while the distal part of the long arm was translocated to chromosome 21. The female patient had severe Potter syndrome stigmata and died at 4.5 hours of age. In addition, short stature and excessive skin at the neck, compatible with Turner syndrome, were found. It was not surprising, since the patient had only one X chromosome. *SRY* and important parts of the *AZF* regions must have been missing explaining the female sex.

In two of the three men with a 46,XX karyotype the *SRY* gene was found translocated to one of the X chromosomes. In the third case, *SRY* was not detected. The *SRY* gene usually act through a cascade of other activated factors, and male development, in case the *SRY* gene is missing, might be due to for example increased expression of *SOX9*, which is the next link in the cascade usually activated by *SRY*. Thus, mice missing *SRY* will develop into males, if *SOX9* is duplicated [3]. Investigation of *SOX9* in our third man with 46,XX karyotype could not be performed, as he was lost for follow up.

The low height found in the two men with pure 45,X blood karyotypes and in the two men with mosaicism and ring Y chromosome and three cell lines, respectively, may be due to the presence of only one copy of the SHOX gene in all or some of the cells. These men also had *Cubitus valgus*, which is not surprising since SHOX also has a distinct influence on the skeletal phenotype, including height and elbow structure [48]. Since the pseudoautosomal region 1 at the distal end of the sex chromosome are not subjected to inactivation, the expression of SHOX, the most important gene for linear growth, is positively correlated to the numbers of sex chromosomes, although the expression of SHOX is much higher in 46,XY males than in 46,XX females [49]. In another Danish study, it was found that normal height is often not obtained in 45,X/46,XY mosaicism—even after treatment with growth hormone [50].

In this study, neither abnormalities of the heart and kidneys, nor autoimmune diseases were detected in the men with a pure 45,X karyotype and in men with mosaicism for 45,X. This is in contrast to a study where five of 10 males with 45,X/46,XY mosaicism were found to have a bicuspid aortic valve and four (of 10) a dilated or mildly dilated ascending aorta [51]. Bicuspid aortic valves is usually found in about 25% of Turner syndrome females and in only 1% in the background population [52]. A contributing explanation to the difference in cardiovascular abnormalities might be selection bias in that study [51], since the patients were recruited retrospectively, and many denied to participate in the study. Hypothyroidism in males with 45,X/46,XY has been reported in a few cases [53]. Contributing reasons to the different prevalence of abnormalities in various studies might be a very modest number of patients with 45,X/46,XY mosaicism in each study, and that abnormalities connected to the 45,X cell line may have been compensated by the simultaneously present 46,XY cell line.

### AZF microdeletions

While larger Y chromosome deletions can be detected by karyotyping, AZF microdeletions can only be detected by using primers to specific regions of the Y chromosome or by chromosomal array. While the SRY gene, which determines whether the gonad will develop into a testis [6] and the SHOX gene, which is important for growth [54] are located on the short arm of the Y chromosome, the AZF regions are located on the long arm of the Y (Yq) [55].

The *DAZ* gene was described for the first time by Reijo et al*.* [11]. Later the AZF regions: AZFa, AZFb and AZFc were detected [55]. The common markers sY254 and sY255 located in the AZFc region are specific for the DAZ region [56].

The prevalence of AZF microdeletions differs in different ethnic groups, with the frequency being lower in the northern part of Europe compared to that in South and East Europe, Asia, Australia, and North and South America, where prevalences of up to 12% can be detected in men with non-obstructive azoospermia [7]. Here, we found a missing AZF region in 6.1% (53/865), including a frequency of 4% (34/843) AZF microdeletions in azoospermic men with at least one structurally intact Y chromosome.

Microdeletions in the AZFa region are the most severe category, causing azoospermia and SCOS. AZFb microdeletions also cause azoospermia, but histological examination of testicular biopsies may reveal arrested spermatogenesis / maturation arrest [7]. The AZFc microdeletions are the most frequent AZF microdeletion, making up the majority of all Y microdeletions. AZFc microdeletions are less severe than AZFa and AZFb microdeletions, and a low number of sperm can be found in the semen of approximately half the patients with an isolated AZFc microdeletion [7].

Maymon et al. [57] found the Sertoli cells to be mature (CK-18 negative) in nine men with AZF microdeletions of different sizes and localisations suggesting the defect affects the germ cell line directly rather than via a malformation of the Sertoli cells. The DAZ protein was missing in AZFc microdeletions, resulting in variable impairment of the spermatogenesis [57], whereas RNA Binding Motif (RBM) protein was missing in AZFb microdeletions, which in some studies [58, 59] has been suggested to cause spermatocyte arrest.

Based on more extensive analyses of Yq markers it is obvious that AZF deletion may vary in size [10]. These differences may explain differences in histological patterns detected in testicular biopsies. Thus, we found hypospermatogenesis in two men with a partial AZFb deletion, while SCOS was found in three men with a probably complete AZFb deletion. Further studies supplemented with measurements of levels of for example DAZ or RBM proteins may address this hypothesis.

Although the majority of genes on the Y chromosome seem related to reproduction, genes in the AZFa and AZFb regions are expressed in many tissues throughout the male body [60]. Particularly AZFb deletions have also been suggested to be associated with blood pressure [61, 62] and neuropsychiatric functions [63]. However, the role of Y-linked genes in disease warrants further studies.

Since the father only has the one and only Y chromosome to transmit to his sons, the couples should be informed about this before treatment. They should also be aware that the male infertility issues will likely to be transmitted to the next generation.

### Strengths of the study

It is a major strength of the current study that all men in the cohort were collected consecutively, went through the same examination programme, and all examined by the same clinician. The occurrence of the different causes of azoospermia are in line with other studies. A frequency of 13.9% men with a 47,XXY karyotype (of which 0.9% are mosaic karyotypes) is in accordance with previous studies. Thus, frequencies of 10-15% men with Klinefelter syndrome are expected among men with azoospermia [15]. Furthermore, typically 10-15% of Klinefelter syndrome men show a 47,XXY mosaicism [64]. Therefore, this almost unselected population of azoospermic men seems to be real-world data representative for azoospermic men in the general population.

### Limits of the study

Although the cohort was collected prospectively, the study can be considered retrospective since it did not aim to analyse Y chromosome loss at the start of inclusion of men in the cohort. Therefore, we are not allowed to contact the initial patients for further clinical information, including missing values such as height and weight for some patients. Additionally, in most cases, only karyotypes performed on peripheral blood samples were available. However, since Y chromosome loss is presumed to occur more frequently in blood, it would have been useful if a standard chromosome analysis had also been performed on cell types such as fibroblasts, which represent one of the two additional germ layers. Due to ethical causes, gonad histology was not available in all patients. The suggested prevalence of the different genetic abnormalities are imprecise since these conditions are rare, and ideally a control group should have been included. However, since we do not have a control group of healthy men, we used the group of men with pathogenic *CFTR* mutations and CBAVD, shown to have 100% normal spermatogenesis [12], as controls when evaluating testicular volumes and FSH levels in men with Y chromosome loss.

## Conclusions

This study shows that a relatively high frequency of loss of Y chromosome material (6.1%) is present in men with azoospermia. The conditions vary from small microdeletions to complete loss of the Y chromosome. The study confirms that structural abnormal Y chromosomes are unstable and therefore often lost. Men with 45,X cell lines are often small in stature, which may be due to a reduced number of SHOX genes. Abnormalities of the heart and kidneys or autoimmune diseases were not detected in any of the 11 men with a 45,X cell line. It is already well known that testicular histological patterns are nearly associated with deletions in the respective AZFa, b and c regions, and the results of this study suggests that partial AZFb deletions may have milder impact on spermatogenesis compared to complete AZFb deletions.

## Data Availability

Background data are available from the corresponding author upon reasonable request.

## Abbreviations

AMH: anti-müllerian hormone
AZF: azoospermia factor
CBAVD: congenital bilateral absence of vas deferens
CFTR: cystic fibrosis transmembrane conductance regulator
CT: computer tomography
DAZ: deleted-in-azoospermia
EAA: European Academy of Andrology
EMQN: European Molecular Genetics Quality Network
FISH: fluorescence in situ hybridization
FSH: follicular stimulating hormone
LH: luteinizing hormone
RBM: RNA binding motif
RUS: renal ultrasonography
SCOS: Sertoli cell only syndrome
SHOX: short stature homeobox
SOX3: SRY-box transcription factor 3
SOX-9: SRY-box transcription factor 9
SRY: sex-determining region Y
TESE: testicular sperm extraction
TSH: thyroid stimulating hormone
TTE: transthoracic echocardiography
