# Correction: Complete or partial loss of the Y chromosome in an unselected cohort of 865 non-vasectomized, azoospermic men

**DOI:** 10.1186/s12610-023-00218-7

**Published:** 2024-01-05

**Authors:** J. Fedder, C. Fagerberg, M. W. Jørgensen, C. H. Gravholt, A. Berglund, U. B. Knudsen, A. Skakkebæk

**Affiliations:** 1 Centre of Andrology & Fertility Clinic, Odense University Hospital, Kløvervænget 23, 5000 Odense, Denmark; 2 Department of Clinical Medicine, University of Southern Denmark, Odense, Denmark; 3 Fertility Clinic, Horsens Hospital, Horsens, Denmark; 4 Department of Clinical Genetics, Odense University Hospital, Odense, Denmark; 5 Department of Clinical Genetics, Lillebaelt Hospital, Vejle, Denmark; 6 Department of Molecular Medicine, Aarhus University Hospital, Aarhus, Denmark; 7 Department of Endocrinology, Aarhus University Hospital, Aarhus, Denmark; 8 Department of Clinical Medicine, Aarhus University, Aarhus, Denmark; 9 Department of Clinical Genetics, Aarhus University Hospital, Aarhus, Denmark

**Correction: Basic Clin. Androl. 33, 37 (2023)**

**https://doi.org/10.1186/s12610-023-00212-z**

Following publication of the original article [1], the authors reported a typesetting error in the **Discussion** section. The heading **45,X and 46,XX males** was mistakenly written as X45,X and 46,XX males. The publishers apologize for this typesetting error.

The original article [1] has been corrected.
